# Postoperative outcomes in distal hypospadias: a meta-analysis of the Mathieu and tubularized incised plate repair methods for development of urethrocutaneous fistula and urethral stricture

**DOI:** 10.1007/s00383-019-04523-z

**Published:** 2019-08-01

**Authors:** Hans Winberg, Einar Arnbjörnsson, Magnus Anderberg, Pernilla Stenström

**Affiliations:** 1 Department of Pediatric Surgery, Skåne University Hospital, 221 85 Lund, Sweden; 2 Department of Clinical Sciences, Pediatrics, Lund University, Lund, Sweden

**Keywords:** Meta-analyses, Hypospadias repair, Mathieu (PBF), Tubularized incised plate repairs (TIP), Boys, Outcome, Urethrocutaneous fistula, Urethral stricture

## Abstract

**Purpose:**

To compare the two major complications, namely postoperative urethrocutaneous fistula and urethral stricture, between the Mathieu and tubularized incised plate (TIP) repair methods for distal hypospadias.

**Methods:**

In this meta-analysis, electronic databases were searched for comparative studies on the two techniques. The Oxford Centre for Evidence-based Medicine Levels of Evidence was used to evaluate the included studies. The main outcome measure was the frequency of postoperative fistula and urethral stricture. RevMan 5.3 was used for statistical analyses, with *P *< 0.05 indicating statistical significance.

**Results:**

A total of 17 studies, which included 1572 patients, met the inclusion criteria. The frequency of urethrocutaneous fistula did not differ between the Mathieu [115 (13%)] and TIP [90 (13%)] methods [odds ratio (OR) 1.1, 95% confidence intervals (CI) 0.6–1.9; *P* = 0.73)]. Urethral stricture was less frequent after the Mathieu [15 (2%)] method than after the TIP [37 (5%)] method (OR 0.5, 95% CI 0.3–0.8; *P* < 0.01), even after the subgroup analysis of eight randomized controlled trials was included. Overall, the quality of the included studies was determined to be satisfactory. The levels of evidence on which this review was based ranged from 1b to 2b using the CEBM Levels of Evidence.

**Conclusion:**

Compared with TIP repair, Mathieu repair for hypospadias had a significantly lower risk for urethral stricture; however, the risk for urethrocutaneous fistula was similar.

**Electronic supplementary material:**

The online version of this article (10.1007/s00383-019-04523-z) contains supplementary material, which is available to authorized users.

## Introduction

The widely practiced and established procedures to correct distal penile hypospadias are the Mathieu technique with a perimeatal-based flap and tubularized incised plate (TIP) urethroplasty. The Mathieu technique was first described in 1932 and has been mainly used for coronal and subcoronal hypospadias [1]. Tubularized incised plate urethroplasty was first described in 1994 and has been used to correct distal hypospadias [2, 3]. In both the Mathieu technique and TIP urethroplasty, the neourethra is tunneled into the glans to create a vertical meatus, with the aim of making the glans shape and external meatus appearance natural after repair [4]. For both methods, complication rates have been reported to be 2–13% [3–5]. The most common reported serious complications after hypospadias surgery are urethral stricture and urethrocutaneous fistula, both of which require surgical treatment. To date, there has been no consensus on the better choice between the Mathieu and TIP techniques, as well as on the short- and long-term outcomes of both procedures [5]. This meta-analysis aimed to compare the postoperative rates of urethrocutaneous fistula and urethral stricture between the Mathieu technique and TIP urethroplasty.

## Methods

### Search strategies

The meta-analysis was conducted following the PRISMA guidelines [6]. Using the keyword “hypospadias,” all literature published from January 1990 to January 2019 was searched in PubMed, Embase, and Cochrane databases. The inclusion criteria were “hypospadias,” “Mathieu,” “tubularized incised plate repairs,” “TIP,” “Snodgrass,” and “complications” data that could be obtained from the paper. Cases were included only if the complications were identified and described with clarity in the paper. Filters were set for articles in English and those that included different age groups (i.e., infants, children, and adolescents).

First, all the abstracts were screened; all the studies that reported postoperative complications as an outcome after the Mathieu and TIP techniques were considered to meet the inclusion criteria. Then, the full articles were retrieved. All the eligible abstracts and articles were assessed independently by HW and EA for inclusion in the meta-analysis.

### Inclusion criteria

This study included all comparative studies who reported on urethrocutaneous fistula and urethral stricture after hypospadias repair by the two repair methods, the Mathieu and TIP techniques on boys younger than 18 years of age.

### Exclusion criteria

This study excluded all non-original articles; those with cohorts smaller than ten patients; those with a greater than 10:1 ratio between the two techniques; those that lacked reports on the two complications studied; those that reported overlapping data; and those in previously published articles. To reduce the risk of modification of the methods that might have influenced the rate of complications, studies with time interval of greater than 20 years were excluded. Studies that included repeat operations were included when differentiating between repeat and primary interventions was not possible.

### Complications

Complications were defined according to Clavien–Dindo [7]; studies with grade 3b complications of urethrocutaneous fistula and urethral stricture that required reoperation under general anesthesia were included. The definition of stricture was subjectively decided by the surgeon/author or was objectively measured in correlation with the patients age or postoperative time. Complications, such as infections and wound dehiscence, as well as cosmetics, were excluded from the analyses.

### Data extraction

The data extracted from the included articles were the study characteristics, such as authors, publication year, sample size, time span, surgical technique (Mathieu or TIP technique), and follow-up period, and patient characteristics, including age at surgery and degree of hypospadias. Specific information on the postoperative complications was collected and analyzed. In cases of uncertainty, events were not included.

### Quality assessment

The level of evidence and publication type was classified according to the Oxford CEBM [8].

### Statistical analysis

The Mantel–Haenszel method was used to calculate pooled odds ratio (OR) [9]. Dichotomous variables were analyzed by estimating the ORs with 95% confidence intervals (CIs). *P* values < 0.05 were considered statistically significant. The RevMan 5.3 statistical package was used to conduct the meta-analysis [10].

## Results

**Fig. 1:**
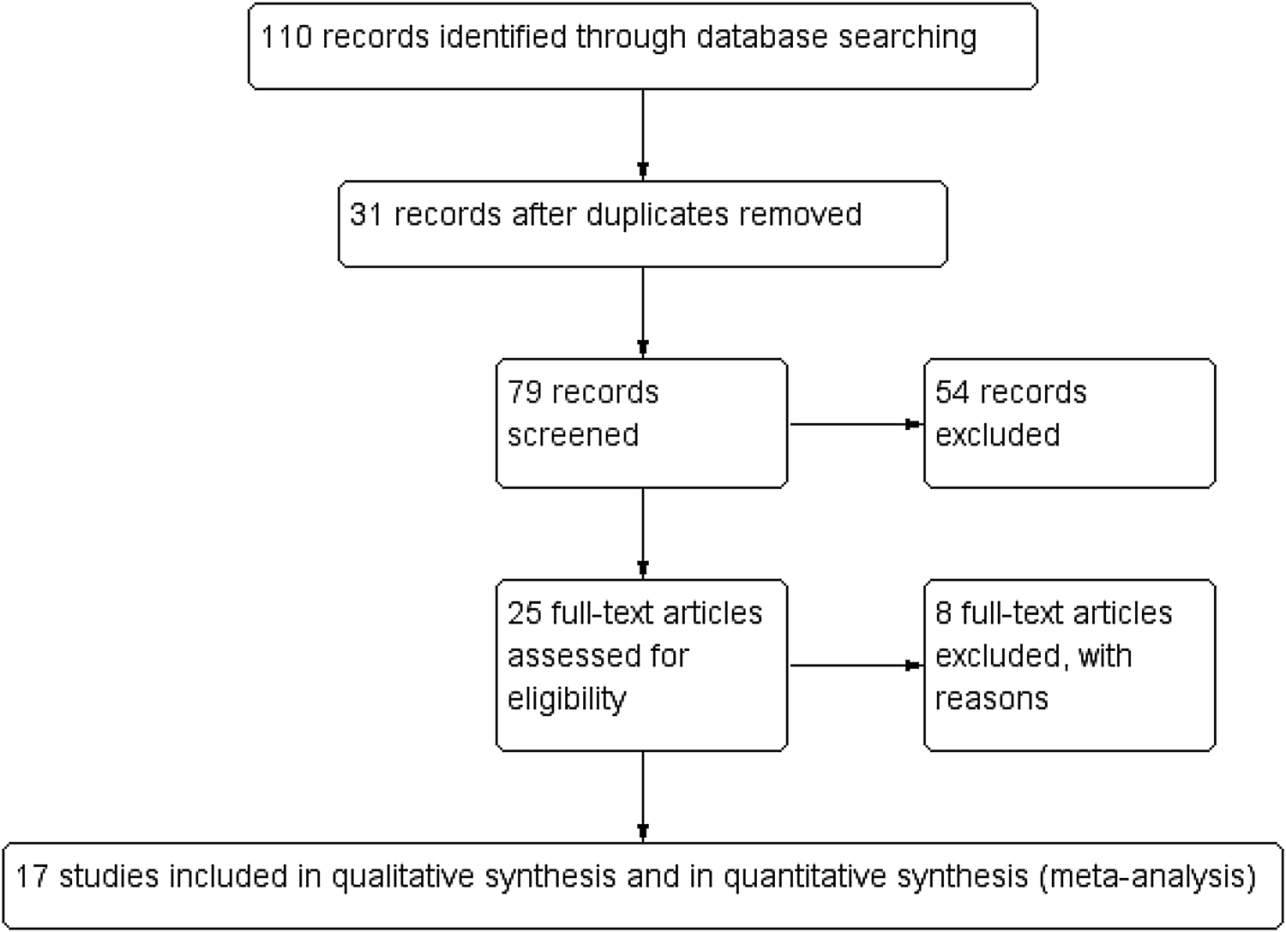
Flowchart of the process to search for articles that compared the complications after hypospadias reconstruction using the Mathieu and TIP repair methods

A search for “hypospadias, Mathieu, TIP repair, and children” provided 110 studies, of which 17 were relevant for this study. A total of 110 abstracts were screened, of which 17 studies met the eligibility criteria. After collecting the information from the full text articles, all 17 studies fulfilled the criteria to be included in the final meta-analysis [11–27] (Fig. 1). Eight of the analyzed studies were RCTs [11, 15–17, 20–22, 24] (Table 1). All the included RCTs included comparisons between Mathieu and TIP. The overall CEBM criteria ranged from 1b to 2b. Search for hypospadias and meta-analysis revealed seven publications, but none of those were relevant for this study.

**Table 1:** A summary of the studies included in this meta-analysis

References	Year	Level of evidence	Mathieu	TIP	Mathieu		TIP	
			*n*	*n*	Fistulae	Stricture	Fistulae	Stricture
Aminsharifi [11]	2008	1b^a^	20	20	0 (0%)	0 (0%)	2 (10%)	5 (25%)
Anwar-ul-haq [12]	2006	2b^b^	45	45	7 (16%)	3 (7%)	3 (7%)	2 (4%)
Bae [13]	2014	2b	13	25	1 (8%)	0 (0%)	6 (24%)	1 (4%)
Chrzan [14]	2007	2b	25	26	1 (4%)	1 (4%)	17 (65%)	1 (4%)
Elganainy [15]	2010	1b	64	37	5 (8%)	0 (0%)	3 (8%)	5 (14%)
Guo [16]	2004	1b	43	36	11 (26%)	1 (2%)	3 (8%)	2 (6%)
Hamid [17]	2014	1b	48	52	6 (13%)	4 (8%)	3 (6%)	3 (6%)
Imamoğlu [18]	2003	2b	54	56	4 (7%)	2 (4%)	4 (7%)	5 (9%)
Karabulut [19]	2008	2b	9	4	5 (56%)	0 (0%)	2 (50%)	1 (25%)
Moradi [20]	2005	1b	18	15	1 (6%)	0 (0%)	2 (13%)	1 (7%)
Nezami [21]	2010	1b	33	21	1 (3%)	0 (0%)	1 (5%)	1 (5%)
Oswald [22]	2000	1b	30	30	2 (7%)	1 (3%)	0 (0%)	0 (0%)
Oztorun [23]	2017	2b	331	161	38 (11%)	1 (0%)	23 (14%)	1 (1%)
Samore [24]	2006	1b	10	10	2 (20%)	1 (10%)	2 (20%)	1 (10%)
Ugras [25]	2006	2b	34	20	5 (15%)	0 (0%)	3 (15%)	1 (5%)
Winberg [26]	2016	2b	69	73	24 (35%)	0 (0%)	10 (14%)	4 (5%)
Yildiz [27]	2010	2b	16	79	2 (13%)	1 (6%)	6 (8%)	3 (4%)
		Sum	862	710	115	15	90	37

*TIP* tubularized incised plate

^a^Randomized controlled trial (RCT)

^b^Cohort study

### Study characteristics

A total of 1572 patients (range, 13–492 patients per study) were included in the meta-analysis. Of these, 862 (55%) had undergone surgery with the Mathieu technique and 710 (45%) with TIP technique. Some local variations in the surgical methods were noted during the data extraction, although the general principles of the methods were equal and allowed comparison. Data on age at surgery, weight, and indication for the hypospadias repair were not provided in detail in all the studies; therefore, these were not included in the current analysis. A summary of the characteristics of the included studies is shown in Table 1.

### Frequencies of fistula and stricture

Overall, 259 of 1572 (16%) boys developed complications of fistulas and urethral strictures that required reoperation. Specifically, these complications occurred in 130 of 862 (15%) patients who underwent the Mathieu procedure and in 129 of 710 (18%) patients who underwent the TIP procedure.

### Urethrocutaneous fistula

**Fig. 2:**
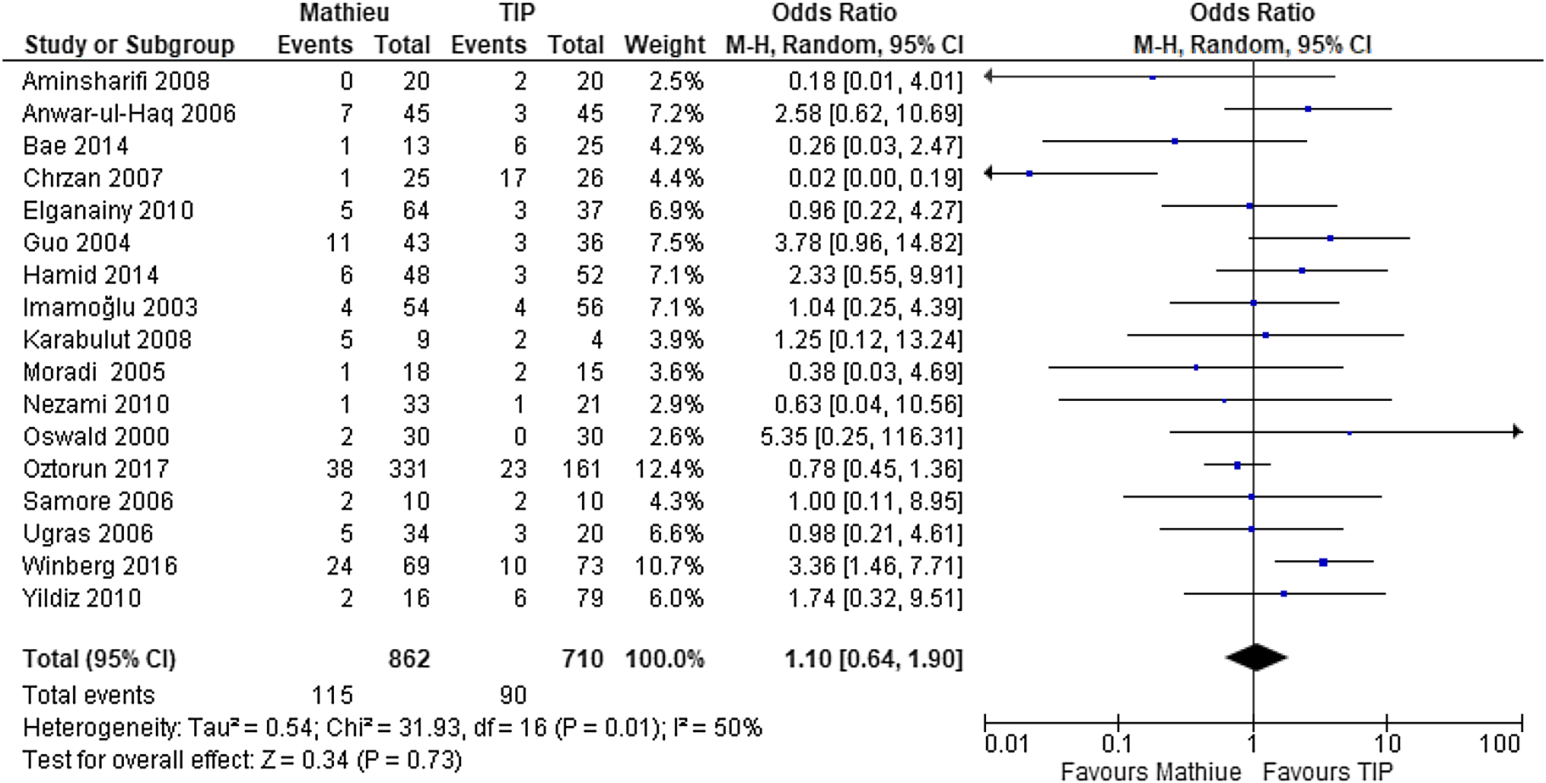
Forest plot of the comparison between the Mathieu and TIP repair methods for hypospadias reconstruction, in terms of fistula formation

In all the 17 studies, postoperative urethrocutaneous fistula was assessed in 1572 patients [11–27]. Fistula was the most common observed and reported complication in 13% (205/1572) of all studies and was equally distributed between the Mathieu and TIP techniques (13% each) without any significant difference between the two methods (Fig. 2). The frequency of postoperative fistula formation varied between 0 and 56% with Mathieu repair and between 0 and 65% with the TIP technique. Meta-analysis of the 17 studies revealed that the risk of fistula development after the Mathieu procedure did not significantly differ from that after the TIP procedure (OR 1.1, 95% CI 0.6–1.8; *P* = 0.82) (Fig. 2).

### Urethral stricture

**Fig. 3:**
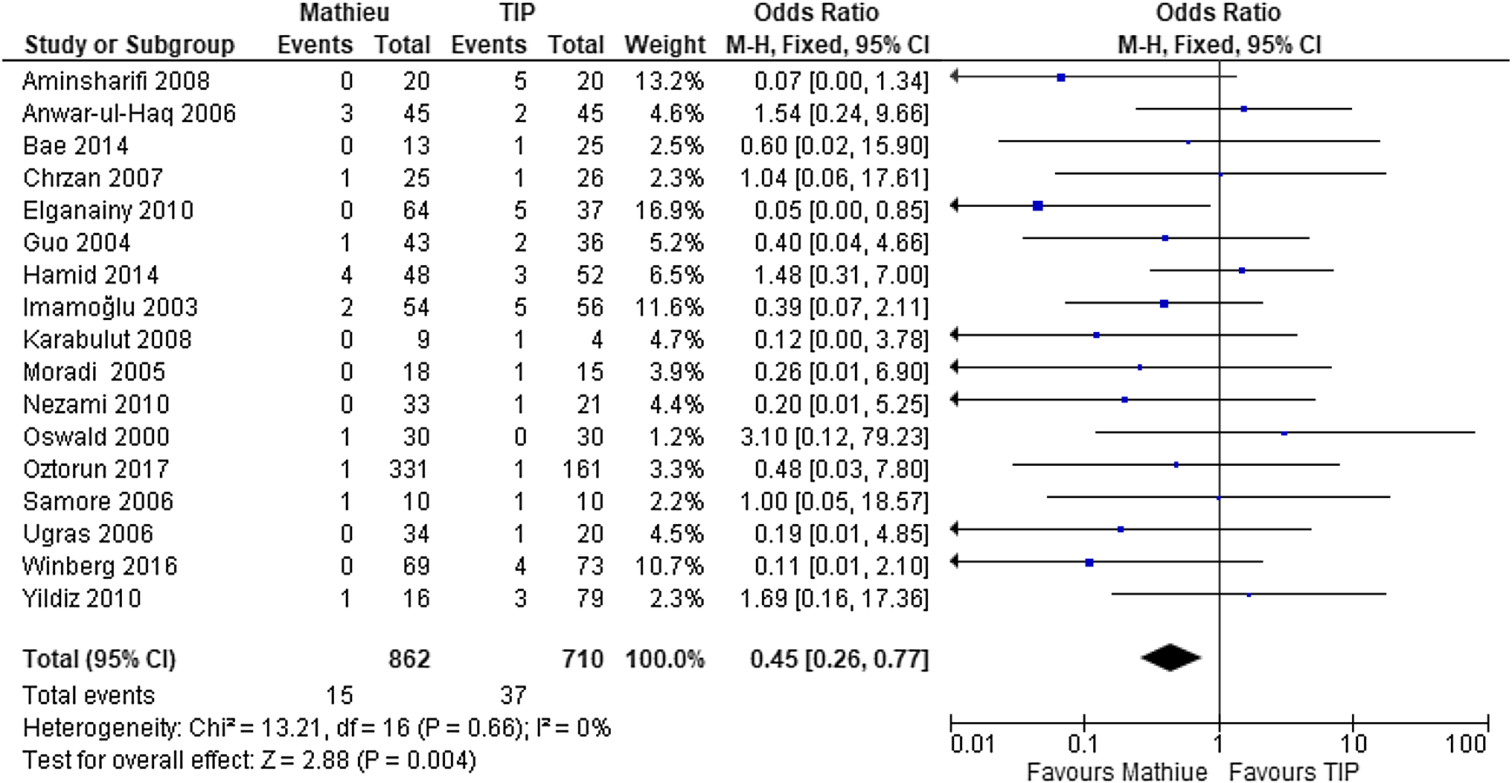
Forest plot of the comparison between the Mathieu and TIP repair methods for hypospadias reconstruction, in terms of postoperative strictures

Urethral stricture was evaluated in all the 17 studies [11–27]. The risk for stricture complications was 0–10% after the Mathieu procedure and 0–25% after the TIP procedure. In most reports, a clear definition of the urethral stricture was missing. After pooling the data of the 17 studies, the rate of urethral stricture appeared to be significantly lower after the Mathieu (2%) than after TIP (5%) (OR 0.5, 95% CI 0.3–0.8; *P* = 0.004) (Fig. 3).

### Analysis of randomized controlled trials

**Fig. 4:**
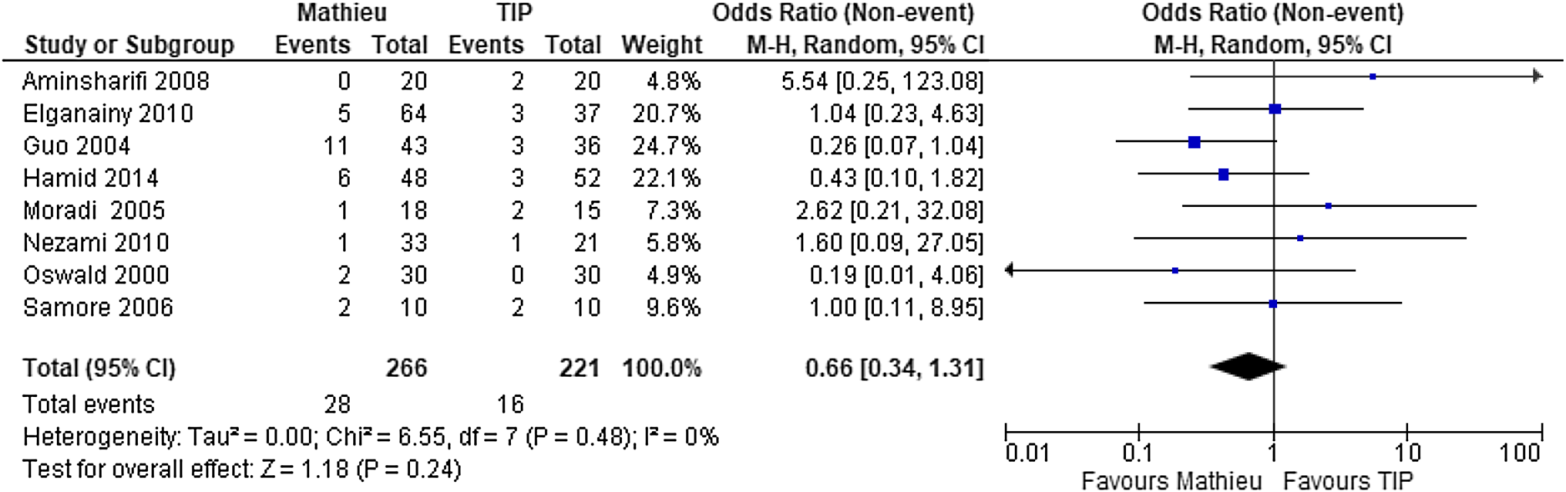
Forest plot of the comparison between the Mathieu and TIP repair methods for hypospadias reconstruction, in terms of fistula formation among the RCTs

**Fig. 5:**
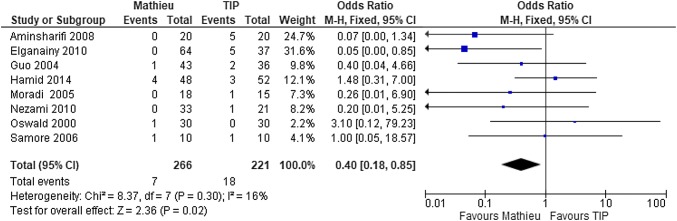
Forest plot of the comparison between the Mathieu and TIP repair methods for hypospadias reconstruction, in terms of postoperative strictures among the RCTs

The methodological quality of the RCTs was analyzed. In both the overall and subgroup (RCTs) [11, 15–17, 20–22, 24] analyses, the rate of fistula did not differ between the two methods (Fig. 4), but the rate of stricture was significantly lower after the Mathieu than after the TIP technique (*P* = 0.02) (Fig. 5).

### Consistency

The details of the surgical procedures varied in some of the analyzed studies. The Mathieu technique was sometimes slightly modified, and the coverage of the neourethra was performed by either a single-layer or double-layer technique. Furthermore, there were diverse variations in the size and material of the suture used for urethroplasty and glansplasty and in the size of the stent, if used, for urinary diversion. Excluding the three publications that contained some repeat procedures [17, 18, 22] did not change the results on the absence of differences between the two methods in terms of urethrocutaneous fistulas and on the lower frequency of strictures after the Mathieu repair than after the TIP technique.

### Publication bias

Possible publication bias was analyzed by reviewing the distribution of the results on funnel plot diagrams (Figures 6–9) found in the supplementary material. The funnel plot figures demonstrated only some asymmetry, suggesting little evidence of publication bias.

## Discussion

This meta-analysis was based on 17 reports that included 1572 patients. The level of evidence ranged from 1b to 2b. Fistula was the most common (13%) complication after hypospadias repairs and had a similar rate between the Mathieu and TIP techniques. Both techniques had low rates of postoperative complications, but the risk for urethral stricture was lower after the Mathieu than after TIP. Subgroup analysis of eight RCTs showed consistent results.

To our best knowledge, this was the first meta-analysis that showed significant differences in postoperative complications between the Mathieu and TIP techniques for hypospadias repair. Similar to this study, one systematic review (2012) and another meta-analysis (2013) showed that both the Mathieu and TIP repair methods had low rates of postoperative complications (2–15%), and no significant difference was found overall [5, 28]. A systematic review of studies published before 2010 concluded that both reconstruction techniques for distal hypospadias had low rates of postoperative complications, but it did not present a clear consensus on the ideal method [5]. However, that study was not included in this present meta-analysis, because of a CEBM level of evidence of only four [8].

The first report on TIP dates to 1994; therefore, including 1990 through 1994 is unnecessary. Excluding these years from the search does not change the findings in this study. Over the years, TIP repair has evolved numerous times as can be seen by following Snodgrass publications [29–32]. Long TIP is not so common, and the use of tunica vaginalis has helped decrease fistula formation. Excluding the oldest repairs, the incidence of the fistula formation and urethral stricture may drop off dramatically. Classifying the findings of the study according to the historical milestones of the TIP technique evolution and demonstrating the outcomes accordingly would be of great interest. This is, however, not feasible due to the small series reported and cannot be done without increasing the bias in the study.

Many factors may influence the repair of hypospadias; therefore, comparison and analysis of literature may not be precise. The analyzed studies did not always document or consistently report on the presence of a ventral curvature, surgeons experience, patients age, previous penile surgery, quality of the penile skin, and configuration of the corpora. The quality of the urethra is an important factor. If there is divergence of the corpus spongiosum, the patient is not a candidate for Mathieu but might be a candidate for TIP. Detailed information is missing in the included reports. It has been documented that a dartos flap reduces the risk of fistula with TIP [33]. Patients or studies that did not include a dartos flap were not excluded due to inconsistent reporting of this in the included publications. A significant complication in TIP is glans dehiscence. Including this complication in the study when evaluating distal hypospadias repair results would add significantly to the paper. However, these data are not available and not consistently reported throughout all the included publications. Therefore, in this meta-analysis, adjustment for confounding factors was not possible, and we could not take full account of the many variables that are related to fistula formation.

Urethral stricture was the second most common complication after hypospadias surgery, with a rate of 2% after the Mathieu procedure and 5% after the TIP procedure. Our pooled data estimated a higher rate of urethral stricture for TIP than for the Mathieu technique. As in line with fistulas, possible causes for strictures could not be analyzed further because of the lack of information about the confounding factors.

The complications of wound dehiscence and wound infection were excluded from this study. These parameters were not consistently defined or reported in the studies collected for the meta-analysis. Besides, these complications may precede or be a part of the two primary outcomes studied, i.e., fistula and stricture. In the meta-analysis, the secondary outcomes, such as cosmetic results and functional outcomes, were not evaluated. Evaluating these secondary long-term outcomes is necessary in future studies for the comparison of those two techniques and was not in the scope of this study.

In the absence of any functional outcomes, such as uroflows, it is necessary to have some reservation to accept the conclusions of the study, which states that the Mathieu technique, because of the lower risk for postoperative urethral stricture, may be the preferred method for hypospadias. Urinary flow measurements were not consistently reported throughout the literature studied. It is important to note that the long-term result of the Mathieu hypospadias repair is associated with a hairy urethra [34] and abnormal uroflows.

Our study had several limitations. Some critical data were rarely documented, making it impossible to adjust for all possible confounding factors. The significant clinical heterogeneity made our conclusions somewhat conservative, although we used a random effects model. Furthermore, there was a lack of uniform criteria for reporting outcomes and we assumed that the criteria were similar enough to be assessed together. The lack of late follow-up re-stricture is another limitation. Therefore, future studies with complete data and uniform criteria are needed, to identify the best operative intervention for hypospadias. Nevertheless, this study provided valuable information and up-to-date information in this field. The level of evidence on which our review was based was high and our conclusion differed from that of some previous reports [5, 28]. Our results are in favor of the Mathieu over the TIP technique, regarding lowering the risk for urethral stricture.

### Risk of bias

There was potential bias in the review process, including selection bias due to random sequence generation and allocation concealment. Furthermore, performance bias cannot be excluded due to the binding of personnel, and detection bias might be present due to the binding of outcome assessment. Incomplete outcome data (attrition bias), selective reporting (reporting bias), or publication bias was likewise possible.

## Conclusions

Compared with TIP urethroplasty, the Mathieu technique for hypospadias reconstruction was associated with a significantly lower risk for postoperative urethral stricture and may be the preferred method for hypospadias. However, the clinical implications of the results can be discussed because the risk of stricture in both procedures was low. The implications for research are obvious because there had been no studies that provided firm guidelines on the best method for the operative intervention for hypospadias.

## Electronic supplementary material

Below is the link to the electronic supplementary material.

Supplementary material 1 (DOCX 36 kb)
