# Supplementary material for: Postoperative outcomes in distal hypospadias: a meta-analysis of the Mathieu and tubularized incised plate repair methods for development of urethrocutaneous fistula and urethral stricture
Source: Pediatr Surg Int. 2019 Aug 1;35(11):1301–8. doi: 10.1007/s00383-019-04523-z (PMC6800881; doi:10.1007/s00383-019-04523-z)

**Figure 6** Comparison of fistula formation between the Mathieu and TIP methods for hypospadias repair in 17 studies


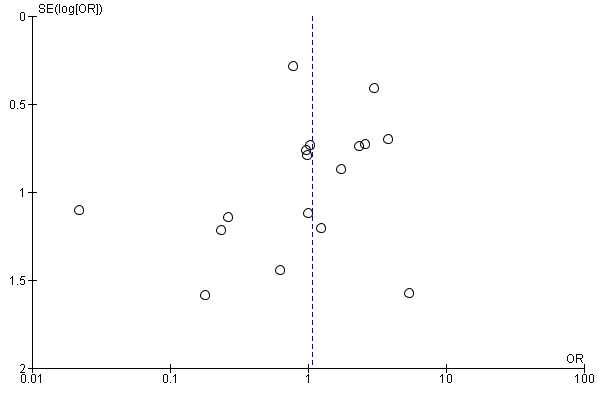


**Figure 7** Comparison of postoperative strictures between the Mathieu and TIP methods for hypospadias repair in 17 studies


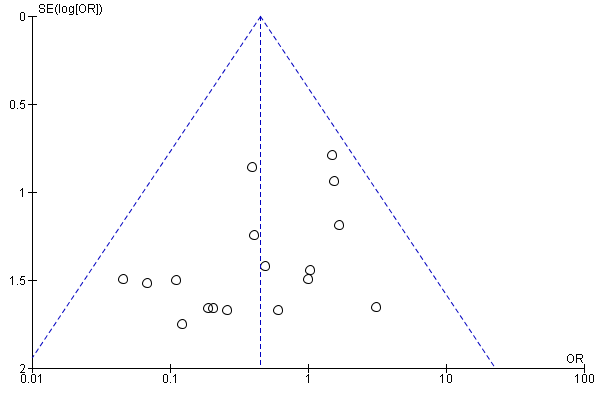


**Figure 8** Comparison of fistula formation between the Mathieu and TIP methods for hypospadias repair in 8 randomized controlled studies


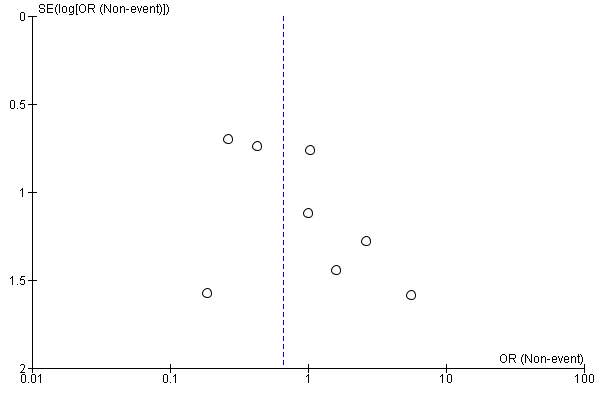


**Figure 9** Comparison of postoperative strictures between the Mathieu and TIP methods for hypospadias repair in 8 randomized controlled studies


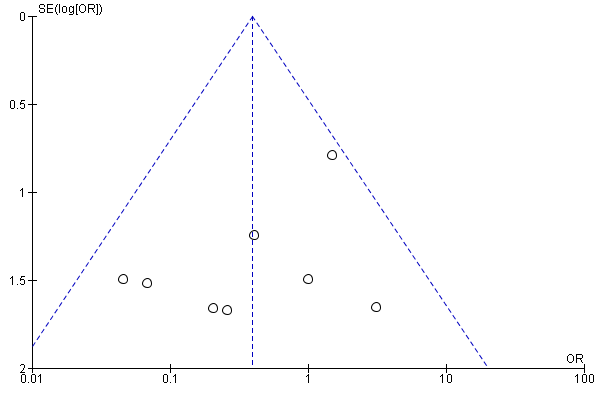

Supplement: Supplementary file 1 — Supplementary material 1 (DOCX 36 kb) [file 383_2019_4523_MOESM1_ESM.docx]
